# Tinnitus Patients with Comorbid Headaches: The Influence of Headache Type and Laterality on Tinnitus Characteristics

**DOI:** 10.3389/fneur.2017.00440

**Published:** 2017-08-28

**Authors:** Berthold Langguth, Verena Hund, Michael Landgrebe, Martin Schecklmann

**Affiliations:** ^1^Department of Psychiatry and Psychotherapy, University of Regensburg, Regensburg, Germany; ^2^Department of Psychiatry, Psychosomatics and Psychotherapy, kbo-Lech-Mangfall-Klinik Agatharied, Hausham, Germany

**Keywords:** migraine, cluster headache, trigeminus, phantom sound, laterality, comorbidity

## Abstract

**Background:**

Both clinical experience and clinical studies suggest a relationship between tinnitus and headache. Here, we aimed to investigate the influence of comorbid headache type and headache laterality on tinnitus characteristics.

**Method:**

The Tinnitus Research Initiative database was screened for patients of the Tinnitus Center of the University Regensburg who reported comorbid headaches. These patients were contacted to complete additional validated questionnaires. Based on these data, patients were categorized according to headache type and headache laterality, and their clinical characteristics were compared with tinnitus patients, who did not report comorbid headaches.

**Results:**

Data from 193 patients with tinnitus and comorbid headaches were compared with those from 765 tinnitus patients without comorbid headaches. Tinnitus patients with comorbid headache have higher scores in tinnitus questionnaires, a lower quality of life and more frequently comorbidities such as painful sensation to loud sounds, vertigo, pain (neck, temporomandibular, and general), and depressive symptoms when compared with tinnitus patients without headaches. Both headache laterality and headache type interact with the degree of comorbidity with higher impairment in patients with left-sided and bilateral headaches as well as in patients with migraine or cluster headache.

**Conclusion:**

The observed increased impairment in tinnitus patients with comorbid headache can be explained as an additive effect of both disorders on health-related quality of life. The more frequent occurrence of further comorbidities suggests a generally increased amplification of sensory signals in a subset of tinnitus patients with comorbid headaches.

## Introduction

Tinnitus, the perception of sound in the absence of a corresponding sound source, is a frequent disorder. In some forms of tinnitus, there is an internal sound source like sounds from abnormal blood flow because of vascular anomalies or palatal myoclonus. These forms are defined as objective tinnitus. In contrast, in subjective tinnitus, there exist neither external nor internal sound sources. Subjective tinnitus can vary in its perceptual characteristics (loudness, pitch, number of tones, tonal or noise-like, pulsatile vs. non-pulsatile), its laterality (unilateral, bilateral, in the head), its maskability, its etiology, and its comorbidities. Accordingly, it has been assumed that there exist many different forms of tinnitus that also may differ in their pathophysiology. The identification of relevant criteria for subtyping different forms represents a major challenge in tinnitus research (1, 2). Specific comorbidities such as hyperacusis (3) or temporomandibular joint (TMJ) disorders (4) have turned out to represent potentially relevant criteria for subtyping. As an example patients with tinnitus and comorbid TMJ disorders were younger, more frequently female and suffered from less hearing loss (4), indicating that this group represents a clinically relevant tinnitus subtype.

Moreover, comorbidities such as hyperacousis (3), hearing loss (5), insomnia (6, 7), depression (8, 9), and pain syndromes (10) play a major role for tinnitus-related impairment in quality of life. Tinnitus-related health burden can be measured by specific validated tinnitus questionnaires (TQs) like the TQ (11) or the tinnitus handicap inventory (12), but also by numeric rating scales (13).

In the previous studies, an association between tinnitus and headaches has been demonstrated (14–16). These studies indicate that between 26 and 47% of patients with tinnitus also suffer from headache. Particularly frequent among tinnitus patients are unilateral headache syndromes (16). Since unilateral headaches and unilateral tinnitus symptoms occur in the majority of cases on the same side and headache and tinnitus are interacting over time, alterations in the trigeminal nerve activity have been proposed as a potentially relevant overlapping pathophysiological factor (16). Based on this reasoning, one may assume that headache as a comorbidity may represent a relevant factor for subtyping of tinnitus.

In order to investigate comorbid headache as potential criterion for tinnitus subtyping, we retrospectively analyzed clinical data from patients who presented at the multidisciplinary Tinnitus Center at the University of Regensburg. Patients who reported the existence of headaches in the Tinnitus Sample Case History Questionnaire (TSCHQ) (17) and who completed an additional headache questionnaire (18) were compared in their clinical and demographic characteristics with those patients who had tinnitus without headaches. In detail, we investigated whether tinnitus patients with specific forms or laterality of headache differ in demographic or other clinical characteristics from those tinnitus patients without headaches.

## Materials and Methods

### Sample

The analysis was based on datasets of all patients, who presented at the multidisciplinary Tinnitus Center of the University of Regensburg between 2003 and 2011 and whose data were included in the Tinnitus Research Initiative database (19) (*n* = 1,817). All patients who reported the existence of headaches in the TSCHQ [answer “yes” to the question “Do you suffer from headaches?” (17)] (*n* = 489) were contacted by mail and asked to complete additional questionnaires (16). In these additional questions, patients were asked about the laterality of tinnitus and headache (Is your headache on one or predominantly one-sided? Is your tinnitus on one or predominantly one-sided?) and about the relationship between tinnitus and headache (time of onset of tinnitus and headache, respectively, and interaction between tinnitus and headache intensity). Completed headache and TQs were obtained from 193 patients, and this sample was compared with patients from the database who answered “no” to the question “Do you suffer from headaches?” in the TSCHQ (*n* = 765). Please note that three patients did not indicate the site of the headaches and were not included for the statistical analyses of headache laterality. Informed consent was obtained from all patients participating in the study. The study was approved by the Ethic committee of the University of Regensburg (11-101-0286), and all data were pseudonymized for analysis.

To exclude sample bias, we compared the patients with completed questionnaires with the group of patients with headache who did not respond to the mail (*n* = 235). Both groups did not differ significantly with respect to age at tinnitus onset (*t* = 0.805; df = 395; *p* = 0.421), gender (χ^2^ = 0.566; df = 1; *p* = 0.452), tinnitus duration (*t* = 0.693; df = 395; *p* = 0.489), tinnitus distress as indicated by TQ (*t* = 0.114; df = 405; *p* = 0.909) and tinnitus handicap inventory (*t* = 0.072; df = 413; *p* = 0.943), and mean hearing threshold (*t* = 0.513; df = 304; *p* = 0.608).

### Assessment of Headaches and Tinnitus Severity

For the classification of headaches, the headache questionnaire by Fritsche et al. (18) was used, which was developed and validated according to the 2nd version of the classification criteria of the International Headache Society. The questionnaire enables to differentiate migraine, tension headache, cluster headache, combination of migraine and tension headache, and combination of tension- and cluster headache and non-classifiable headache.

Clinical and demographic information was obtained from available data of the investigated patients in the TRI database (19). Available data included the TSCHQ (17), the TQ (11), the Tinnitus Handicap Inventory (20, 21), various numeric tinnitus rating scales (13), the Beck depression inventory (BDI) (22), and the WHO Quality of Life Questionnaire (23).

### Statistical Analysis

The relationship between the different demographic and clinical characteristics of tinnitus and the existence of comorbid headache, its laterality and its type was analyzed by using chi-square tests for categorical clinical variables and with analyses of variance (ANOVAs) for metric clinical variables. *Post hoc* tests were done for significant effects using least significant difference tests for ANOVAs and adjusted residuals (*z* > 1.96 was indicated as significant) for the chi-square tests and are indicated in Tables 1 and 2.

**Table 1:** Influence of headache laterality on sample characteristics.

	Headache left	Headache right	Headache bilateral	No headache	Statistics
*N*	49	43	98	765	n.a.
Age at tinnitus onset (years)	44.8 ± 15.3 (*n* = 47)	42.4 ± 11.9 (*n* = 41)	41.6 ± 13.3 (*n* = 89)	44.0 ± 14.3 (*n* = 715)	*F* = 0.937; df = 3,888; *p* = 0.422
Duration of tinnitus (months)	89.9 ± 85.3 (*n* = 47)	100.7 ± 119.2 (*n* = 41)	97.5 ± 118.6 (*n* = 89)	100.7 ± 106.4 (*n* = 714)	*F* = 0.165; df = 3,887; *p* = 0.920
**Gender (female/male)**	**22/27^a^**	**22/21^a^**	**31/67**	**208/557^a^**	**χ^2^ = 17.413; df = 3; *p* < 0.001**
**Tinnitus questionnaire**	**47.4 ± 19.7 (*n* = 46); >no**	**40.6 ± 18.3 (*n* = 40)**	**46.9 ± 16.8 (*n* = 96) >no**	**38.8 ± 17.2 (*n* = 736)**	***F* = 10.964; df = 3,914; *p* < 0.001**
**Tinnitus handicap inventory**	**54.3 ± 23.8 (*n* = 46); >no**	**48.8 ± 24.2 (*n* = 42)**	**56.2 ± 21.9 (*n* = 97); >no**	**44.0 ± 22.3 (*n* = 737)**	***F* = 10.983; df = 3,918; *p* < 0.001**
**Beck depression inventory**	**14.2 ± 9.8 (*n* = 44); >no**	**12.0 ± 9.1 (*n* = 42); >no**	**14.5 ± 8.8 (*n* = 96); >no**	**9.1 ± 7.4 (*n* = 731)**	***F* = 19.545; df = 3,909; *p* < 0.001**
Numeric rating scale loudness	6.7 ± 2.5 (*n* = 44)	6.5 ± 1.8 (*n* = 40)	6.8 ± 2.1 (*n* = 98)	6.2 ± 2.2 (*n* = 726)	*F* = 3.262; df = 3,904; *p* = 0.021
**Numeric rating scale discomfort**	**7.6 ± 2.0 (*n* = 43); >no**	**7.0 ± 2.2 (*n* = 41)**	**7.6 ± 2.1 (*n* = 97); >no**	**6.6 ± 2.3 (*n* = 725)**	***F* = 7.241; df = 3,902; *p* < 0.001**
Numeric rating scale annoyance	7.1 ± 2.4 (*n* = 43)	6.7 ± 2.7 (*n* = 40)	7.1 ± 2.2 (*n* = 98)	6.4 ± 2.4 (*n* = 728)	*F* = 3.402; df = 3,905; *p* = 0.017
Numeric rating scale unpleasentness	6.9 ± 2.3 (*n* = 44)	6.7 ± 2.6 (*n* = 40)	7.1 ± 2.2 (*n* = 98)	6.4 ± 2.4 (*n* = 728)	*F* = 2.858; df = 3,906; *p* = 0.036
Numeric rating scale ignorability	7.0 ± 2.6 (*n* = 44)	7.0 ± 2.8 (*n* = 41)	7.0 ± 2.4 (*n* = 98)	6.6 ± 2.7 (*n* = 728)	*F* = 0.872; df = 3,907; *p* = 0.455
**WHO quality of live—physical health**	**13.6 ± 2.9 (*n* = 33); <no**	**14.3 ± 3.0 (*n* = 27); <no**	**13.4 ± 3.0 (*n* = 66); <no**	**15.5 ± 2.6 (*n* = 515)**	***F* = 16.543; df = 3,537; *p* < 0.001**
**WHO quality of live—psychological factors**	**13.0 ± 3.6 (*n* = 33); <no**	**14.0 ± 2.8 (*n* = 28)**	**13.3 ± 2.7 (*n* = 66); <no**	**14.7 ± 2.6 (*n* = 417)**	***F* = 8.243; df = 3,540; *p* < 0.001**
WHO quality of live—social relationships	14.2 ± 3.7 (*n* = 33)	13.6 ± 3.7 (*n* = 27)	14.0 ± 2.6 (*n* = 66)	15.0 ± 3.1 (*n* = 417)	*F* = 3.614; df = 3,539; *p* = 0.013
WHO quality of live—environment	15.7 ± 2.4 (*n* = 33)	15.9 ± 2.0 (*n* = 28)	15.7 ± 2.0 (*n* = 65)	16.5 ± 2.1 (*n* = 418)	*F* = 3.916; df = 3,540; *p* = 0.009
Sensitivity to loud sounds	3.4 ± 1.2 (*n* = 47)	3.3 ± 1.2 (*n* = 40)	3.3 ± 1.2 (*n* = 96)	3.2 ± 1.2 (*n* = 744)	*F* = 1.329; df = 3.923; *p* = 0.264
**Painful sensations by loud sounds (yes/no)**	**28/14**	**26/13**	**68/23^a^**	**354/314**	**χ^2^ = 19.053; df = 3; *p* < 0.001**
Pulsatile tinnitus (no/yes with…/yes different from heartbeat)	33/7/7	28/7/5	80/10/6	605/72/64	χ^2^ = 7.863; df = 6; *p* = 0.248
Tinnitusquality (tonal/noise/cricket/other)	25/6/11/4	27/3/7/5	61/14/17/5	449/76/144/72	χ^2^ = 5.686; df = 9; *p* = 0.771
Influence by noise (yes/no)	31/12	32/5	63/21	493/158	χ^2^ = 2.658; df = 3; *p* = 0.447
Somatic modulation (yes/no)	24/24	17/25	42/55	258/486	χ^2^ = 7.045; df = 3; *p* = 0.070
Mean hearing threshold	26 ± 13 (*n* = 44)	19 ± 11 (*n* = 42)	18 ± 12 (*n* = 96)	23 ± 15 (*n* = 731)	*F* = 3.890; df = 3,682; *p* = 0.009
Side of worse hearing (left/left = right/right)	20/2/10	13/2/7	35/5/33	284/35/228	χ^2^ = 2.914; df = 6; *p* = 0.820
**Vertigo (yes/no)**	**28/19^a^**	**23/20^a^**	**52/43^a^**	**158/585^a^**	**χ^2^ = 87.258; df = 3; *p* < 0.001**
**Temporomandibular joint complaints (yes/no)**	**17/31^a^**	**11/31**	**29/68^a^**	**124/620^a^**	**χ^2^ = 19.471; df = 3; *p* < 0.001**
**Neck pain (yes/no)**	**42/7^a^**	**32/10^a^**	**69/29^a^**	**341/402^a^**	**χ^2^ = 56.552; df = 3; *p* < 0.001**
**General pain (yes/no)**	**25/22^a^**	**25/18^a^**	**58/39^a^**	**249/480^a^**	**χ^2^ = 35.427; df = 3; *p* < 0.001**
**Current psychiatric treatment (yes/no)**	**12/36^a^**	**8/32**	**26/70^a^**	**89/659^a^**	**χ^2^ = 21.608; df = 3; *p* < 0.001**

*Bold printed lines indicate significant effects*.

*^a^The number of cases in this cell is significant different from the number of expected cases under the assumption of independence of variables*.

*< or > indicate significant post hoc contrasts*.

*Please note that high values in quality of life mean high quality of life and that high values in the other measures mean high burden*.

**Table 2:** Influence of headache type on sample characteristics.

	Non-classifiable headache	Migraine	Tension-type headache	Tension-type headache and migraine	Cluster headache	No headache	Statistics
*N*	63	86	25	11	8	765	n.a.
Age at tinnitus onset (years)	44.0 ± 14.2 (*n* = 59)	41.4 ± 12.8 (*n* = 81)	41.3 ± 12.5 (*n* = 24)	40.0 ± 17.7 (*n* = 8)	52.1 ± 12.1 (*n* = 7)	44.0 ± 14.2 (*n* = 715)	*F* = 1.260; df = 5,888; *p* = 0.279
Duration of tinnitus (months)	84.7 ± 107.3 (*n* = 59)	104.1 ± 114.1 (*n* = 81)	111.9 ± 119.5 (*n* = 24)	61.0 ± 82.3 (*n* = 8)	94.0 ± 95.9 (*n* = 7)	100.7 ± 106.4 (*n* = 714)	*F* = 0.544; d = 5,887; *p* = 0.743
Gender (female/male)	19/44	33/53	13/12^a^	6/5	5/3^a^	208/557^a^ (*n* = 715)	χ^2=^18.761; df = 5; *p* = 0.002
**Tinnitus questionnaire**	**44.7 ± 18.2 (*n* = 60); >no**	**45.8 ± 18.4 (*n* = 81); >no**	**45.8 ± 17.7 (*n* = 24); >no**	**38.5 ± 14.7 (*n* = 11); <cluster**	**56.6 ± 15.4 (*n* = 8); >migraine + tension; >no**	**38.0 ± 17.2 (*n* = 736)**	***F* = 6.561; df = 5,914; *p* < 0.001**
**Tinnitus handicap inventory**	**52.0 ± 23.4 (*n* = 61); >no**	**54.9 ± 23.2 (*n* = 84); >no**	**55.5 ± 23.2 (*n* = 25); >no**	**43.9 ± 21.3 (*n* = 10)**	**61.9 ± 21.7 (*n* = 8); >no**	**44.0 ± 22.3 (*n* = 737)**	***F* = 6.379; df = 5,919; *p* < 0.001**
**Beck depression inventory**	**12.5 ± 8.4 (*n* = 59); >no**	**14.8 ± 9.2 (*n* = 83); >no**	**12.9 ± 10.3 (*n* = 25); >no**	**11.8 ± 8.5 (*n* = 10)**	**17.0 ± 10.4 (*n* = 8); >no**	**9.1 ± 7.4 (*n* = 731)**	***F* = 11.813; df = 5,910; *p* < 0.001**
Numeric rating scale loudness	6.5 ± 2.3 (*n* = 59)	6.8 ± 2.1 (*n* = 84)	7.0 ± 2.1 (*n* = 25)	7.0 ± 2.1 (*n* = 10)	6.6 ± 2.2 (*n* = 7)	6.2 ± 2.1 (*n* = 726)	*F* = 1.973; df = 5,905; *p* = 0.080
**Numeric rating scale discomfort**	**7.1 ± 2.2 (*n* = 60)**	**7.6 ± 2.1 (*n* = 83); >no**	**8.0 ± 1.8 (*n* = 24); >no**	**7.3 ± 2.3 (*n* = 10)**	**7.0 ± 1.6 (*n* = 7)**	**6.6 ± 2.3 (*n* = 725)**	***F* = 4.593; df = 5,903; *p* < 0.001**
Numeric rating scale annoyance	6.9 ± 2.4 (*n* = 59)	7.0 ± 2.3 (*n* = 83)	7.7 ± 2.2 (*n* = 25)	6.7 ± 2.5 (*n* = 10)	6.2 ± 2.2 (*n* = 7)	6.4 ± 2.4 (*n* = 728)	*F* = 2.406; df = 5,906; *p* = 0.035
Numeric rating scale unpleasentness	6.9 ± 2.2 (*n* = 59)	7.0 ± 2.4 (*n* = 84)	7.3 ± 2.5 (*n* = 25)	6.6 ± 2.4 (*n* = 10)	6.3 ± 2.2 (*n* = 7)	6.4 ± 2.4 (*n* = 728)	*F* = 1.728; df = 5,907; *p* = 0.126
Numeric rating scale ignorability	6.8 ± 2.3 (*n* = 60)	7.3 ± 2.7 (*n* = 84)	6.9 ± 2.7 (*n* = 25)	6.4 ± 2.6 (*n* = 10)	6.1 ± 2.2 (*n* = 7)	6.6 ± 2.7 (*n* = 728)	*F* = 0.961; df = 5,908; *p* = 0.441
**WHO quality of live—physical health**	**13.4 ± 3.3 (= 44); <no**	**13.5 ± 3.0 (*n* = 56); <no**	**14.8 ± 2.1 (*n* = 19)**	**15.7 ± 1.8 (*n* = 4)**	**12.8 ± 1.8 (*n* = 6); <no**	**15.5 ± 2.6 (*n* = 415)**	***F* = 10.764; df = 5,538; *p* < 0.001**
**WHO quality of live—psychological factors**	**13.5 ± 3.4 (*n* = 45); <no**	**13.2 ± 2.8 (*n* = 56); <no**	**13.8 ± 2.6 (*n* = 19)**	**15.8 ± 0.7 (*n* = 4); >no**	**12.3 ± 2.4 (*n* = 6); <tension + migraine; < no**	**14.7 ± 2.6 (*n* = 417)**	***F* = 5.556; df = 5,541; *p* < 0.001**
WHO quality of live—social relationships	14.3 ± 3.7 (*n* = 44)	13.8 ± 2.8 (*n* = 56)	14.4 ± 3.1 (*n* = 19)	14.7 ± 2.9 (*n* = 4)	11.6 ± 3.5 (*n* = 6)	15.0 ± 3.1 (*n* = 417)	*F* = 2.940; df = 5,540; *p* = 0.012
WHO quality of live—environment	15.9 ± 2.4 (*n* = 45)	15.5 ± 1.9 (*n* = 55)	16.6 ± 1.6 (*n* = 19)	15.1 ± 1.1 (*n* = 4)	14.4 ± 2.3 (*n* = 6)	16.5 ± 2.1 (*n* = 418)	*F* = 3.635; df = 5,541; *p* = 0.003
Sensitivity to loud sounds	3.1 ± 1.3 (*n* = 61)	3.5 ± 1.2 (*n* = 83)	3.2 ± 1.1 (*n* = 24)	3.1 ± 1.6 (*n* = 10)	4.0 ± 0.9 (*n* = 8)	3.2 ± 1.2 (*n* = 744)	*F* = 1.929; df = 5,924; *p* = 0.087
**Painful sensations by loud sounds (yes/no)**	**38/21**	**57/20^a^**	**15/6**	**9/1^a^**	**5/3**	**354/314^a^**	**χ^2=^21.061; df = 5; *p* < 0.001**
Pulsatile tinnitus (no/yes with…/yes different from heartbeat)	50/4/7	60/15/7	20/3/2	9/1/0	4/2/2	605/72/64	χ^2=^13.263; df = 10; *p* = 0.209
Tinnitusquality (tonal/noise/cricket/other)	40/11/8/4	52/9/16/6	14/4/5/1	6/0/3/1	2/0/3/3	449/76/144/72	χ^2=^19.358; df = 15; *p* = 0.198
Influence by noise (yes/no)	43/14	58/16	17/7	7/1	3/1	493/158	χ^2=^1.193; df = 5; *p* = 0.946
Somatic modulation (yes/no)	26/37	42/42	8/17	3/7	6/2	258/486	χ^2=^13.751; df = 5; *p* = 0.017
Mean hearing threshold	18.5 ± 12.3 (*n* = 47)	21.3 ± 13.4 (*n* = 55)	18.9 ± 10.5 (*n* = 16)	16.4 ± 6.7 (*n* = 8)	30.5 ± 9.1 (*n* = 7)	22.8 ± 14.5 (*n* = 555)	*F* = 1.807; df = 5,682; *p* = 0.109
Side of worse hearing (left/left = right/right)	25/2/107	28/6/20	8/0/8	3/1/4	5/0/1	284/35/228	χ^2^ = 7.197; df = 10; *p* = 0.639
**Vertigo (yes/no)**	**29/31^a^**	**52/33^a^**	**12/12^a^**	**5/6**	**6/2^a^**	**158/585^a^**	**χ^2=^91.328; df = 5; *p* < 0.001**
**Temporomandibular joint complaints (yes/no)**	**21/41^a^**	**25/59^a^**	**5/20**	**3/8**	**4/4^a^**	**124/620^a^**	**χ^2=^22.777; df = 5; *p* < 0.001**
**Neck pain (yes/no)**	**48/14^a^**	**62/24^a^**	**19/6^a^**	**9/2^a^**	**8/0^a^**	**341/402^a^**	**χ^2=^58.133; df = 5; *p* < 0.001**
**General pain (yes/no)**	**31/30^a^**	**52/34^a^**	**15/10^a^**	**7/3^a^**	**4/4**	**249/480^a^**	**χ^2=^36.522; df = 5; *p* < 0.001**
**Current psychiatric treatment (yes/no)**	**11/50**	**28/55^a^**	**4/21**	**1/9**	**2/6**	**89/659^a^**	**χ^2=^30.493; df = 5; *p* < 0.001**

*Bold printed lines indicate significant effects*.

*^a^The number of cases in this cell is significant different from the number of expected cases under the assumption of independence of variables*.

*< or > indicate significant post hoc contrasts*.

*Please note that high values in quality of life mean high quality of life and that high values in the other measures mean high burden*.

All analyses were performed with SPSS (Statistical Package for Social Studies, Version 19; SPSS Inc., USA). All tests were performed as two-sided tests, and the level of significance was set at 0.001 to correct for the fact that 56 comparisons were performed.

## Results

For evaluation of clinical and demographic characteristics of patients with tinnitus and headaches, patients with left-sided, right-sided, and bilateral headaches were compared with patients with tinnitus, but without headaches from the TRI database (see Table 1).

About 27% of the tinnitus patients in our database answered “yes” to the question “do you suffer from headaches.” Even if comparisons with population-based studies are difficult, as results depend strongly on the method of assessment, a proportion of 27% is not indicative of a substantially altered prevalence of headaches among tinnitus patients.

There was no significant interaction between headache laterality and age at tinnitus onset or tinnitus duration, but a significant interaction between headache laterality and gender, with women suffering more frequently from unilateral headache.

Analyses of tinnitus distress, depressive symptoms, numeric ratings scales, and quality of life showed similar results for all of these measures with increased burden for the group of patients with bilateral and left-sided headaches in contrast to patients with no headaches. The group of patients with right-sided headache showed no difference to the group of patients without headaches or ranged in between the left-sided/bilateral groups and the group with no headaches. This pattern could be seen descriptively in all variables but falling below the significance threshold for TQ, tinnitus handicap inventory, BDI, the numeric rating scale discomfort, and the quality of life domains physical and psychological health. As an exploratory analysis, the TQ score (TQ of the database entry: *r* = 0.321; *n* = 175; *p* < 0.001; TQ by response to mail: *r* = 0.356; *n* = 184; *p* < 0.001) also correlated with the headache frequency (number of days with headache/month).

Painful sensations through loud sounds were more frequently reported by patients with bilateral headache in contrast to patients with unilateral or no headaches. There were no significant effects concerning sensitivity to loud sounds, pulsatile character, tonal versus noise-like character, the ability to mask tinnitus through other sounds, duration of tinnitus, and the ability to modulate tinnitus through neck or jaw movements. There was no significant relationship between headache laterality and hearing function (measured as mean hearing threshold across all frequencies and both sides) nor with the side of worse hearing.

Patients with all headache forms (bilateral, right-sided, and left-sided) reported more frequently comorbid vertigo, TMJ complaints (except right-sided headaches), neck pain as well as pain in general. Patients with headaches were also more frequently treated by psychiatrists (except right-sided headaches). In separate analyses, differences in tinnitus characteristics between patients with specific types of comorbid headaches and no headaches were analyzed. Detailed results are provided in Table 2.

The headache type had no significant influence on age at tinnitus onset nor on tinnitus duration. However, there was a non-significant trend toward an interaction between gender and headache type with women suffering more frequently from comorbid tension-type and cluster headache.

Tinnitus patients with comorbid migraine, tension-type headache, cluster headache, and non-classifiable headache had all higher scores in the TQ, the THI, and the BDI when compared with tinnitus patients without comorbid headaches. Descriptively, highest scores were found for patients with comorbid cluster headache. Patients with combined tension-type headache and migraine did not differ from the other groups except for the TQ showing lower values in contrast to cluster headache.

In the different numeric rating scales, there were statistically significant differences for tinnitus discomfort (higher scores for patients with migraine and tension-type headaches in contrast to patients without headaches), whereas there were no differences for tinnitus severity, unpleasantness, and ability to ignore the tinnitus. The headache type had a significant influence on two domains of the WHOQoL (somatic, psychological) with impairment in quality of life in patients with comorbid unclassifiable headache, migraine, and cluster headaches in contrast to patients without headaches.

Headache type had no influence on the sensitivity to loud sounds, but on the induction of painful sensations by sounds, which was significantly more frequent in patients with migraine and in patients with combined migraine and tension-type headache.

There was no significant influence of headache type on the proportion of patients with hearing function, pulsatile tinnitus, tone or noise-like tinnitus, maskability of tinnitus by environmental sounds, or the ability to modulate tinnitus by somatic maneuvers.

Comorbid headaches had a significant influence on comorbid vertigo, neck pain, TMJ complaints, and pain in general. A higher prevalence of neck pain was found in all headache types and a higher prevalence of vertigo in all headache types apart from combined migraine and tension-type headache. TMJ complaints were more frequent in non-classifiable headache, migraine, and cluster headache, and general pain complaints were more frequent in all patients apart from cluster headache. Patients with comorbid migraine were also more frequently psychiatrically treated.

## Discussion

The main findings of this study are that tinnitus patients with comorbid headache have higher scores in TQs, a lower quality of life and more frequently comorbidities such as painful sensation to loud sounds, vertigo, neck pain, TMJ complaints, general pain, and depressive symptoms when compared with tinnitus patients without headaches. The higher impairment in quality of life in patients who suffer from both tinnitus and headache can be easily explained by a pure additive effect of both disorders on disease burden.

Both headache laterality and headache type interact with the degree of morbidity.

In detail, higher impairment is reported by patients with left-sided and bilateral headaches as well as by patients with migraine or cluster headache. We are aware that there is a certain interaction between headache type and headache laterality, which has to be considered in the interpretation of the results (see Table 3).

**Table 3:** Interaction between headache type and laterality.

	Non-classifiable headache	Migraine	Tension-type headache	Tension-type headache and migraine	Cluster headache
Headache left	14	24	2^a^	3	6^a^
Headache right	11	19	8	3	2
Headache bilateral	36	42	15	5	0^a^

*Chi-square of independence indicates a significant association of headache type and laterality with decreased number of left-sided tension-type and bilateral cluster headache and increased frequency of left-sided cluster headache (χ^2=^17.926; df = 8; *p* = 0.022)*.

*^a^The number of cases in this cell is significant different from the number of expected cases under the assumption of independence of variables*.

*< or > indicate significant post hoc contrasts*.

Our findings are in line with earlier studies, which demonstrated that tinnitus severity is higher in patients with comorbid headache (24) and correlates with headache frequency (25). A potential interaction between tinnitus and migraine (26–30) or other trigeminoautonomal headache syndromes (31, 32) has been described in many studies.

An important question is, whether the co-occurrence of tinnitus and headaches is pure co-incidence or whether there is a pathophysiological interaction. Several potential mechanisms for such a pathophysiological interaction have been proposed. First, increased excitability of the trigeminal system could link tinnitus and headache syndromes (16). Second, central sensitization in the context of migraine could provide an explanation for the development of tinnitus (28). Third, tinnitus could represent a symptom of vestibular migraine (33–35) or vestibular migraine could be related to a specific subtype of Meniere’s disease (36). Fourth, migraine might cause pulsatile tinnitus by vascular alterations during Migraine attacks (30). Fifth, TMJ or neck pain could represent a common cause of comorbid headache and tinnitus. However, due to the cross-sectional design of our study, we can only describe symptom associations and cannot draw any firm conclusions about potential causal interactions between headaches and tinnitus.

In this study, patients with comorbid cluster headache demonstrated highest scores in TQs and most pronounced impairment in quality of life. Induction of painful sensations by loud sounds, general pain syndromes, and psychiatric treatment was most frequent in migraine patients. These findings do not necessarily indicate a specific pathophysiological interaction between headache syndromes and tinnitus, as an increased sensitivity to loud sounds is similar like vertigo a typical feature of migraine. The increased prevalence of psychiatric treatment can be easily explained by the well-known association of headache disorders with anxiety and depression (37). Patients who suffer from tinnitus and headache are more impaired in their quality of life and the higher scores in the TQs may purely reflect a higher health-related handicap, as many questions in the TQ and THI are not tinnitus specific. This fits with the finding that scores were most pronounced in patients with cluster headaches, which are known to be extremely debilitating. The higher comorbidities for pain syndromes, vertigo, sound-induced painful sensations, depressive syndromes, and the higher proportion of psychiatric treatment in this patient group could be explained by a generally increased amplification of sensory signals, which is, for example, encountered in patients with somatoform disorder.

Hints for specific interactions (e.g., a particular high proportion of vertigo in patients with migraine, which could point to vestibular migraine) could not be observed. However, our study cannot exclude that such specific interactions occur, as they can be missed by the statistical analysis, if they occur only in a small group of patients. However, our data also show hints against single additive effects. General pain is not increased in patients with cluster headaches and combination of migraine and tension-type headache did not result in higher burden as indicated by measures of tinnitus distress and discomfort, depressive syndrome, and quality of life.

In addition to headache type also headache laterality had an impact on patients’ characteristics. Comorbid left-sided and bilateral headache had a particular impact on tinnitus severity, on quality of life, and on comorbid disorders such as vertigo, pain, depressive symptoms, and on the frequency of psychiatric treatment. Likewise for headache type, this pattern mainly reflects that patients with comorbid bilateral or left-sided headache are more severely impaired and more frequently suffer also from other somatic, somatoform, and psychiatric symptoms. An earlier analysis of the same sample revealed that left-sided headaches are also frequently associated with left-sided tinnitus and bilateral headaches more frequently with bilateral tinnitus (16). The finding of higher impairment of patients with left-sided symptoms is in line with the literature that shows a slight left-sided preponderance (55–60%) in somatoform disorders (38) and in somatoform pain (39). With respect to headaches, a relatively small study suggests that left-sided migraine is more frequently associated with psychiatric symptoms than right-sided migraine (40). Thus, among tinnitus patients with left-sided headaches, there might be a higher proportion of patients with a somatoform disorder, which can explain the higher impairment in this group. The same explanation may hold true for patients with bilateral headaches, as this group includes also all patients with a rather unspecific description of their headaches, and among patients with rather unspecific description of their headache syndrome a higher proportion of comorbid somatoform disorders is expected as well.

An alternative, but rather speculative explanation for our findings of higher impairment in patients with left-sided symptoms, is provided by a pilot study that investigated the relevance of laterality in able-bodied individuals desiring amputation of a limb (41). In most cases, these individuals desired amputation of a left-sided limb, the disorder was associated with elementary and complex somatosensory disturbances of the affected limb and the most frequent neurological comorbidity was migraine headache. Left-sidedness, limb specificity, and somatosensory disturbances of the affected limb were interpreted as hints for disturbed integration of multi-sensory information of the affected body parts into a coherent cerebral representation of the own body and suggestive of abnormal brain mechanisms in right frontoparietal cortex.

Tinnitus occurs also more frequently on the left side, is tonotopically specific, related to sensory disturbances (42, 43) and an incongruence between visual and auditory input (44), and might therefore be conceptualized as a symptom that compensates an otherwise incoherent cerebral representation of the acoustic environment in relation to the own body.

We are aware of methodological limitations of this study as all data come from one university center and may therefore not be representative. Moreover, data were solely collected by questionnaires and not verified by clinical examination. Thus, further research involving clinical evaluations will be needed to further explore the relationship between different forms of headaches and tinnitus. Nevertheless, our study revealed a greater impairment for tinnitus patients suffering from comorbid headaches and a hint for the occurrence of further comorbidities such as vertigo, pain syndromes, depression, and psychiatric disorders.

## Ethics Statement

This study was carried out in accordance with the recommendations of the ethics committee of the University Hospital Regensburg with written informed consent from all subjects. All subjects gave written informed consent in accordance with the Declaration of Helsinki. The protocol was approved by the ethics committee of the University Hospital Regensburg.

## Author Contributions

VH gathered the data and entered the data in a database. MS performed the statistical analysis. BL and MS drafted the manuscript. All the authors designed the study, interpreted the data, and approved the final version of the manuscript.

## Conflict of Interest Statement

The authors have no conflicts of interest, financial or otherwise, related directly or indirectly to the submitted work. The reviewer, JE-S, and handling editor declared their shared affiliation.
